# Microplastics and exercise: impacts on performance and physiological health

**DOI:** 10.3389/fspor.2025.1611255

**Published:** 2025-08-19

**Authors:** Xianwei Jiao, Qinglei Cao, Zhongyuan Deng

**Affiliations:** ^1^ Physical Education College, Henan Normal University, Xinxiang, China; ^2^ Department of Physical Education, University of Science and Technology Beijing, Beijing, China; ^3^ School of Agricultural Sciences, Zhengzhou University, Zhengzhou, China

**Keywords:** microplastics, athletic performance, healthy environmental pollution, long-term exposure risks, prevention strategies

## Abstract

Microplastics, a widespread and growing environmental pollutant, have raised global concerns due to their pervasive presence in both urban and natural environments. The extensive use of plastics has led to human exposure through inhalation, ingestion, and skin contact, posing potential risks to athletes and fitness enthusiasts during exercise. Studies suggest microplastics may impair exercise performance and health, though research remains limited. Current evidence indicates microplastics enter the body via respiration, water, and food, potentially disrupting physiological functions. However, their exact mechanisms, exposure thresholds, and long-term effects on athletic performance are poorly understood. This paper reviews microplastic sources, exposure pathways in sports, and their physiological impacts, aiming to establish a comprehensive understanding of their role in exercise science. By analyzing existing literature, this study explores microplastics’ influence on physical function, athletic performance, and training outcomes. The findings may help identify actionable prevention strategies, promoting safer sports environments. Further research is needed to clarify microplastics’ health implications and develop effective mitigation measures for athletes.

## 1 Introduction

Microplastics, a type of microplastic particles with a diameter of less than 5 mm, are widely present everywhere in the global environment and have become one of the serious environmental pollution problems (1). Research shows that microplastics are widely present in marine and freshwater resources (2), and more worryingly, they have been proven to penetrate the soil and even the air, posing a serious threat to the balance of the ecological environment and human health (3, 4). Microplastics come from a wide range of sources, including: the natural degradation of plastic products, emissions from industrial processes, and the shedding of synthetic fibers during daily washing and many other aspects (5).

Beyond their general environmental prevalence, microplastics are also widely distributed in sports competition environments. Athletes may be exposed to these contaminants through breathing, inhalation or food ingestion during training and competition. Societal concern over plastic pollution is growing, with particular attention to aquatic environments—an issue that has received widespread media coverage. However, plastic and plas tic byproducts also pollute terrestrial ecosystems, and the potential impacts on organisms are currently poorly understood. Here, we show that a microplastic type used in artificial sport turfs may have negative effects on plant growth. Given the scale of plastic pollution, urgent research is needed to determine the impact of microplastics on terrestrial organisms and their communities, as well as an exploration of alternative, biodegradable materials and measures to reduce the spread of microplastics in nature. Among other factors, characterized by the accumulation of plastic in the environment. While studies on the consequences of plastic pollution for animals, particularly in aquatic environments, have increased in recent years, much less is known about potential effects of plastic pollution on plants in terrestrial environments. Ethylene propylene diene monomer (EPDM) is a microplastic used in artificial sport turfs. Here, we tested in two separate experiments the effects of different concentrations of EPDM on the performance of *Plantago lanceolata* and on competition between seven grassland-plant species. At very low concentrations of the EPDM granules, growth of *P. lanceolata* was slightly improved, but at concentrations of 5% and higher there were strong negative effects on survival and growth. These negative effects were found under low and high nutrient conditions, and for all tested species. The EPDM granules also negatively affected the root weight ratio, which indicates that the root system was more strongly affected than the shoot. Due to the strong negative effects on plant growth, the granules also reduced the competitive interactions between plants. Our study shows that it is not only animals in aquatic environments that may be affected by plastic pollution, and that this may also be the case for wild plants in terrestrial ecosystems (6).

Beyond ecological impacts, the potential threat of microplastics to human health is increasingly a focus of scientific concern—particularly for athletic populations, who are frequently exposed to environments that may contain high levels of such pollutants. These tiny pieces of plastic can enter the human body via a variety of routes, including drinking water, food ingestion, and breathing and inhaling microplastics in the air (7). Their accumulation in the body can cause a series of health problems, such as an enhanced immune system response, endocrine disruption, and a potential increased risk of cancer (8–10). Therefore, it is especially important for people who regularly engage in outdoor activities or sports events involving soil and water bodies to understand and reduce the risk of microplastic exposure. Given the heavy training loads athletes endure and the emerging evidence of microplastic release from specific sports fields (like artificial turf or synthetic tracks), which can be transported via wind or water, athletes face potentially elevated exposure risks, particularly due to the extended time spent in these environments during high-intensity training (11). Study indicates that microplastics can enter the human body through complex food chain pathways, resulting in various health risks. These risks include, but are not limited to, respiratory diseases, immune disorders, and endocrine system imbalances (12). Moreover, microplastics, as a carrier of pollutants, may not only contain harmful chemicals inherently, but also adsorb additional toxic chemical components, thereby exacerbating their potential risks to human health (13). To address these amplified risks, scientists are now delving into the specific mechanisms of action of microplastics on the human body, hoping to formulate and develop effective preventive measures and cleaning strategies for protecting public health.

Within sports activities specifically, microplastics derive from several key sources, amplifying the exposure risk for athletes. These include the soil on the ground layer of the sports field, the water source used for water replenishing, and the wear and tear of sports equipment and apparel worn by athletes during use (14, 15). As the number of athletes continues to rise, the emission and accumulation of microplastics are becoming more and more serious. It is of great practical value and significance to delve into the distribution of microplastics in sports activities and their potential impact on the health of athletes.

Overall, the widespread distribution of microplastics and the health risks they may bring about, especially for athletes, should attract our high attention. Future research should focus on delving into the sources of microplastics specific to sports environments, such as the microplastic particles released by artificial turf during use, the microplastic fibers shed by synthetic sportswear and sports equipment due to wear and tear, and the microplastics possibly present in sports drink packaging and production processes. For athletes, who are a high-risk group for microplastic exposure, it is crucial to thoroughly investigate their unique exposure routes. This includes quantifying the amount of microplastics inhaled by athletes during high-intensity outdoor training, especially in environments with high microplastic concentrations like synthetic turf fields; studying the absorption of microplastics through skin contact when athletes wear synthetic sportswear, which may be exacerbated by friction and sweat during exercise; and analyzing the intake of microplastics by athletes through contaminated food and sports drinks. By gaining a comprehensive understanding of these aspects, future research can provide a solid scientific basis for formulating targeted public health strategies for athletes.

## 2 Sources and characteristics of microplastics

**Figure 1:**
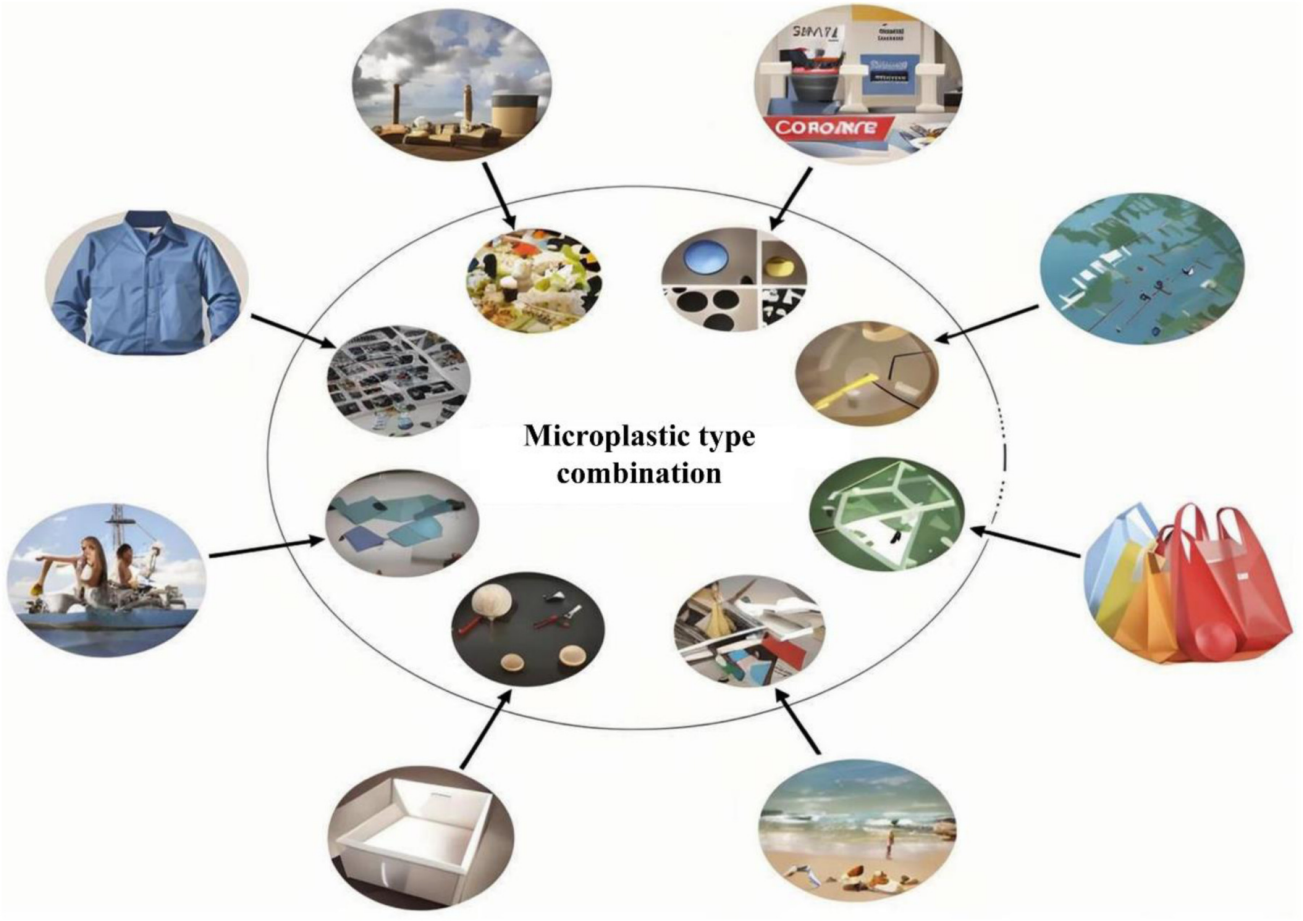
The source of microplastics. The components inside the circle are sourced from microplastics, while the items outside the circle are sourced from microplastics.

Microplastics, defined as microplastics with a diameter of less than 5 mm, are extensively distributed in the environment. Its extensive distribution has two sources. One is microplastic particles directly produced in the manufacturing process of plastic products; the other is secondary microplastics during the gradual decomposition of bulky plastic waste items under the action of the natural environment. These microplastics have a variety of forms and compositions, including fibers, particles, and flakes (16). These microplastics may come from various environments in life, such as inadequate exhaust smoke from factory manufacturing, laboratory plastic supplies, daily plastic bags, beach waste, foam boxes, fiber clothing, etc. (Figure 1). They vary significantly in physical and chemical properties depending on the source and the environment where they are generated or located. Research reveals that microplastics are not only widely distributed in the water and soil environment, but more astonishingly, they can also be transported layer by layer along the food chain and finally enter the living organisms, posing a potential long-term threat to the balance of the ecological environment and human health (17–19).

The main environmental sources of microplastics include the discharge of municipal wastewater, the application of plastic products in agriculture, the decomposition process of plastic commodities, and the pollution caused by marine activities (18). The content of microplastics in urbanized areas is significantly high, especially in urban rivers and lakes, and plastic decomposition and loss are the main sources of pollution (19, 20). In agricultural practice, the use of plastic film and the application of sewage and sludge significantly increase the concentration of microplastics in the soil. Research data show that 33.0% and 29.6% of microplastics are derived from these sources, respectively (21, 22). With the increase in the intensity of human activities, the distribution range of microplastics continues to spread, and microplastics are now widely detected in waters, soils and atmospheres around the world (Table 1).

**Table 1:** Sources of microplastics.

Source type	Specific sources
	Personal care products: Toothpaste, shampoo, shower gel and other products contain plastic microbeads (23).
Primary microplastics	Clothing fibers: Washing synthetic clothes and fabrics (16).
	Industrial raw materials: Plastic pellets and other industrial raw materials (24).
	Daily necessities: Plastic packaging, plastic bags, plastic bottles, etc. (23)
	Agricultural plastics: Farm plastic films, pesticide packaging, etc. (21, 22)
	Marine activities: Marine debris, fishing nets, etc. break down into microplastics due to seawater erosion, UV radiation and mechanical friction (18, 25).
Secondary microplastics	Synthetic fiber products: Sportswear, sports equipment and other synthetic fiber items (26).
	Synthetic turf: Releases microplastic particles as it wears down with use (24).
	Sports drinks: Packaging materials and the production process may release microplastics (25, 27).

Beyond these general environmental sources, microplastics also exhibit distinct patterns in specialized settings—particularly within sports environments. In the context of sports, microplastics show unique distribution patterns and compositional characteristics. Research shows that synthetic materials in sports venues and fitness facilities, especially the synthetic turf, have become one of the important sources of microplastics, and the amount of microplastics they released exhibits a significant upward trend (26). In the sports environment, microplastics are often present in fine particles and variable forms, and they vary in size and shape with the characteristics of surrounding pollution sources. Research also shows that even short-term tourism activities can cause significant fluctuations in the amount and type of microplastics in the beach and its associated sports environment (18, 25). This finding requires us to consider the potential impact of microplastics when designing and managing sports spaces to reduce their negative impact on the environment and health (Table 1).

## 3 Pathways of exposure to microplastics in sports

During sports activities, the human body may be exposed to microplastics through inhalation, direct contact with the skin, ingestion within the food chain, and drinking water and sports beverages containing microplastics (27, 28). A good understanding of these channels is critical to assessing the health risks confronting participants.

**Figure 2:**
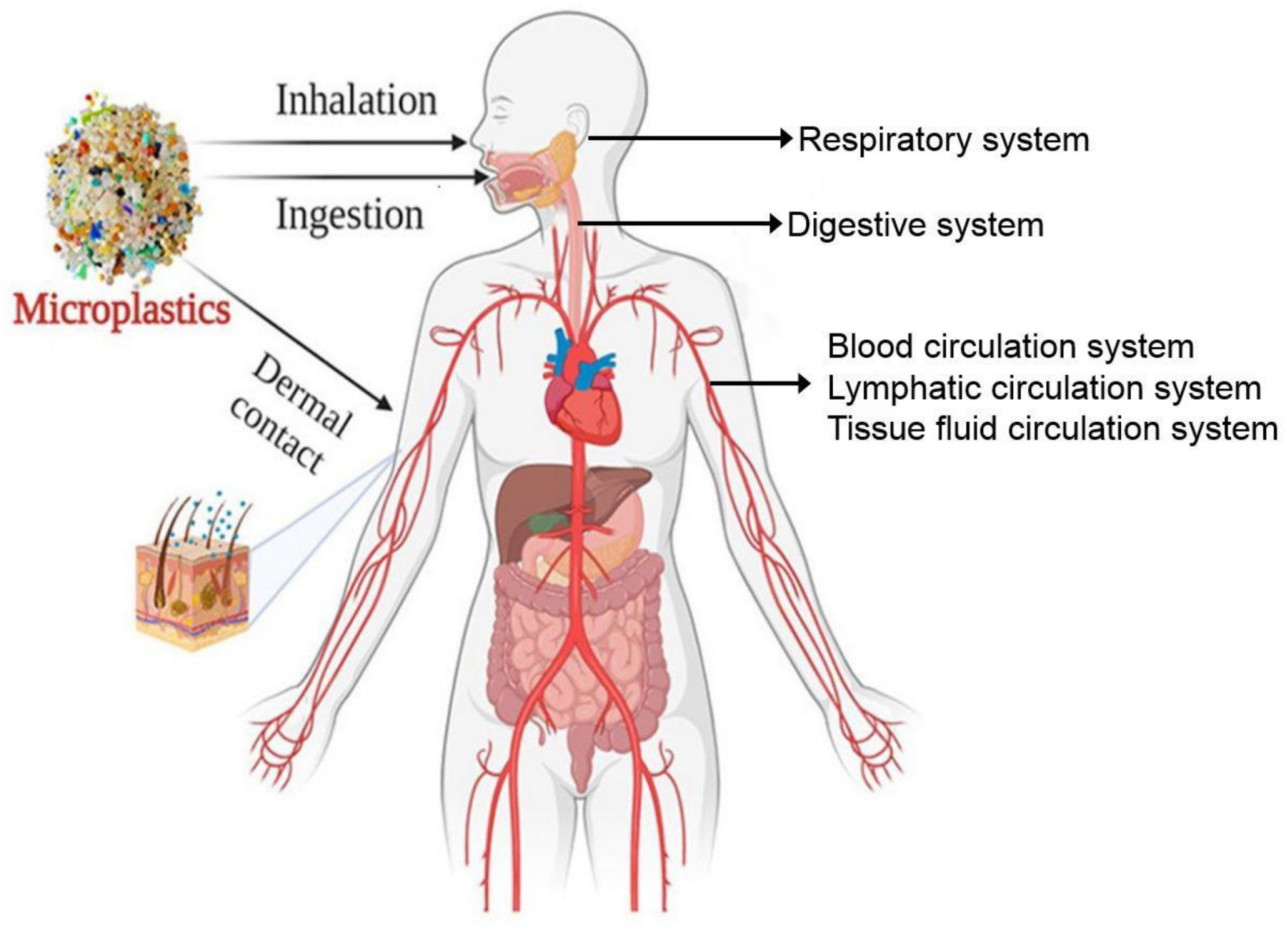
The ways in which microplastics can enter the human body. Mainly include inhalation, ingestion and dermal contact.

During sports activities, especially outdoor high-intensity activities, sports activity participants may inhale microplastic particles contained in the air. These particles may be caused by environmental pollution, decomposition of materials in sports venues, or wear and tear of sports equipment. The microplastics can not only enter the human body through the respiratory system, but also pose a risk of exposure through direct skin contact (Figure 2) (7). For example, some sportswear and accessories may release tiny plastic particles (29) that can even be absorbed through the skin by the body under the action of friction and sweat. Moreover, the inhalation of microplastics may lead to 111 respiratory inflammations and result in cardiovascular diseases, allergic reactions, and other health problems, especially for athletes, whose respiratory rate and depth increase significantly during sports activities, greatly increasing the risk of microplastic inhalation (30).

Widely distributed in water and soil, and easy to transport along the food chain, microplastics pose a potential threat to human health. Athletes, with their high-calorie diets often emphasizing protein-rich sources like seafood and agricultural products, may inadvertently ingest microplastics at elevated rates. These tiny particles, pervasive in oceans and freshwater, are frequently mistaken by aquatic organisms—critical protein sources in many athletes' meal plans—and accumulate up the food chain (31, 32). For instance, competitive swimmers or triathletes relying on seafood for recovery may face higher exposure, as filter-feeding organisms like mussels can accumulate microplastics at concentrations 10,000 times those of surrounding water. Research shows that microplastic ingestion alters physiological processes in commercially farmed fish and shellfish (33), directly impacting the nutritional quality of staples in athletes' diets. In endurance athletes, whose immune systems are already stressed by intense training, microplastic-induced endocrine disruption (34) could exacerbate recovery delays or increase injury susceptibility. Long-term accumulation in athletes—who often maintain strict dietary regimens for years—may compound risks, potentially affecting oxygen transport in blood or muscle repair mechanisms critical to performance. This underscores the need for sport nutrition guidelines to address microplastic contamination in training diets.

Athletes often use sports drinks to replenish water and electrolytes during their exercise. However, recent scientific studies have shown that microplastic contaminants are detected in many sports drink products (35). Data shows that there are approximately 240,000 microplastic particles per liter of beverage on average (36). The sources of these microplastics are likely to be closely associated with the packaging materials and manufacturing processes of the beverages. Athletes may unconsciously ingest microplastics during the process of replenishing water, which has become a health hazard that cannot be ignored. It is urgent to optimize the production and packaging practices for sports drinks for significantly reducing the environmental release from microplastics, and better safeguarding the health and safety of athletes (37).

## 4 Effects of microplastics on exercise physiology

### 4.1 Effects on the respiratory system

**Figure 3:**
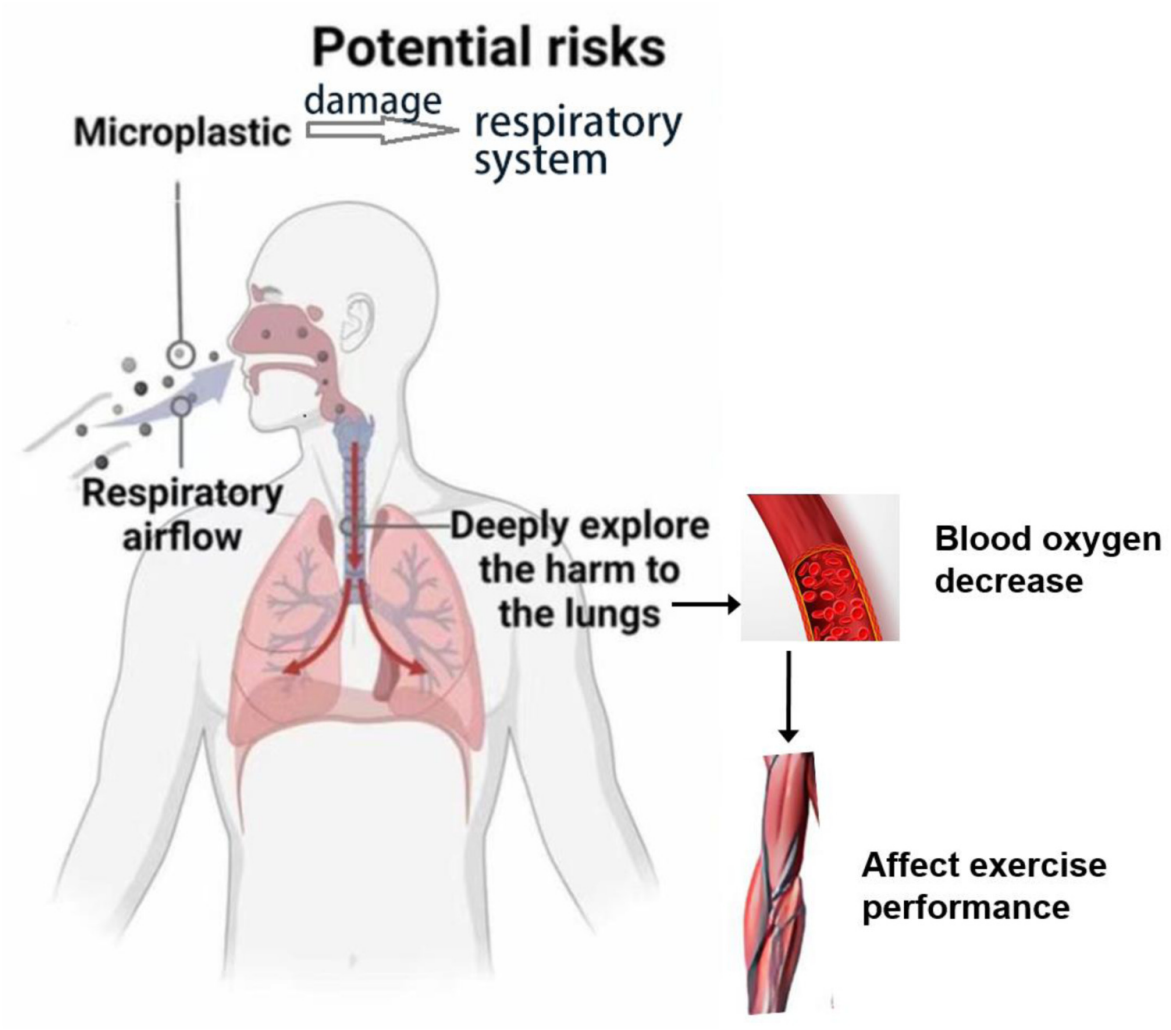
Microplastics affect exercise performance by respiratory system.

The ubiquity of microplastics is attracting global attention, and of particular concern is the potential for them to enter the human body through the air and have long-term effects on the respiratory system- with athletes facing heightened risks due to their unique exposure patterns. Research suggested that the entry of microplastics into the human respiratory tract through inhalation may lead to various health problems, such as lung inflammation, increased oxidative stress and decreased lung function (Figure 3, Table 2). For athletes, this poses specific dangers: endurance runners or cyclists, who inhale 10–20 times more air per minute than sedentary individuals during peak activity, may inhale up to 50% more microplastic particles from polluted air or synthetic turf dust (44). The tiny size of the microplastics allows them to penetrate deep into the lungs and even reach the critical alveolar region—where gas exchange occurs. Research also indicates that the accumulation of microplastics may be associated with the occurrence of chronic respiratory diseases, particularly asthma and chronic obstructive pulmonary disease (COPD) (44, 45). Moreover, microplastics may have long-term effects on the nervous system (Table 2). Microplastics have adverse effects on the cognitive performance through the pathway between the lungs and the brain (lung-brain axis), thus deepening our concern for respiratory health (12, 45). Thus, it is particularly crucial and urgent to investigate the potential effects of microplastics on the respiratory system, especially during physical activities.

**Table 2:** The impacts of microplastics on various human body systems.

System impact	Specific impact	Exercise-related tissues/organs involved
Reproductive System	Affects germ cells, reduces sperm quality and quantity, leads to male reproductive toxicity, affects fetal and ovary development (38, 39).	While not directly exercise-specific, reproductive health indirectly influences overall physical stamina and endurance, which are foundational for sports performance.
Nervous System	Causes nerve cell damage, neuron damage, learning dysfunction, and cognitive impairment (40).	Nerve cells and neurons are critical for motor control, coordination, and reaction time—key components of sports performance and skill execution.
Endocrine System	Leads to liver function damage, lipid metabolism disorders, insulin resistance, and thyroid endocrine disruption (41).	The liver (metabolic regulation), thyroid (energy metabolism), and systems governing lipid/insulin balance directly impact energy production, muscle recovery, and stamina during exercise.
Immune System	Reduces immune function, increases immune cell inflammatory response, causes apoptosis and mitochondrial functional damage (42).	Immune cells and mitochondria in muscle tissues are vital for post-exercise recovery, as impaired immunity increases susceptibility to infections that hinder training, and mitochondrial damage reduces muscle energy output.
Respiratory System	Triggers lung inflammation, oxidative stress, impaired lung function, causing severe harm to the respiratory system (43).	The lungs are directly involved in oxygen uptake during exercise; impaired lung function reduces aerobic capacity, endurance, and overall athletic performance.

### 4.2 Effects on exercise endurance and performance

Intake and inhalation of microplastics may have adverse effects on the exercise endurance and performance. Research shows that exposure to microplastics may reduce the efficiency of oxygen uptake and application during exercise, which affects exercise endurance accordingly (46). In mice experiments, it has been observed that maximum speeds and endurance levels decreased significantly in organisms exposed to microplastics during aerobic exercise (47, 48). Moreover, the presence of microplastics may significantly increase the recovery time after exercise and result in excessive post-exercise oxygen consumption (49). Moreover, the presence of microplastics may significantly prolong post-exercise recovery time and induce excessive post-exercise oxygen consumption (49), which could be attributed to multiple potential mechanisms. Physiologically, microplastics accumulated in the body may trigger systemic low-grade inflammation by activating immune cells such as macrophages (42), disrupting the balance of pro-inflammatory and anti-inflammatory cytokines. This chronic inflammatory state can impair the efficiency of muscle repair—particularly the synthesis of contractile proteins crucial for recovery—and delay the restoration of glycogen stores in skeletal muscles, thereby extending the time required for functional recovery. Additionally, microplastics may interfere with mitochondrial function in muscle cells (50), reducing the efficiency of oxidative phosphorylation and ATP production. This energy metabolism disorder not only exacerbates fatigue during exercise but also hinders the adenosine triphosphate (ATP) supply needed for post-exercise tissue repair, leading to prolonged recovery cycles. Furthermore, microplastics' ability to act as carriers for toxic chemicals (13) adds another layer of complexity. Adsorbed heavy metals or endocrine-disrupting substances could interfere with hormone regulation during recovery, such as disrupting the secretion of growth hormone or testosterone, which are vital for muscle anabolism. For example, endocrine disruption induced by microplastics (41) may alter the body's catabolic-anabolic balance post-exercise, further impeding recovery. These combined effects indicate that microplastics not only interfere with physiological functions during exercise but also compromise post-exercise recovery mechanisms, ultimately undermining exercise performance (51, 52). Therefore, future research should prioritize unraveling these internal mechanisms: exploring how microplastics regulate inflammatory signaling pathways to affect muscle repair, clarifying their impact on mitochondrial bioenergetics during recovery, and investigating interactions between adsorbed toxicants and endocrine systems. Such insights will lay the groundwork for developing targeted interventions—such as anti-inflammatory strategies or mitochondrial protective agents—and provide scientific support for formulating personalized protection plans for athletes and fitness enthusiasts, thereby mitigating the adverse effects of microplastics on exercise performance and recovery.

### 4.3 Effects on muscle functions and recovery

Currently, researchers start to focus on the potential effects of microplastics on muscle functions and recovery. Research shows that the intake of microplastics may trigger the dysfunction of muscle cells and decreased resilience. For example, exposure to microplastics can cause oxidative stress, which can damage the structure and function of muscle cells, and significantly compromise muscle strength and endurance (53, 54). Moreover, microplastics have the potential to interfere with muscle energy metabolism and trigger metabolic disorders during the post-exercise recovery, which has adverse effects on the overall competitive performance of athletes (55). In animal experiment models, organisms constantly exposed to microplastics showed a significant decline in muscle mass and strength, indicating that microplastics may affect its normal functions by interfering with signaling mechanisms within muscle cells (56, 57). In animal experimental models, the specific models referenced in the studies by Amato-Lourenço et al. (56) and Li et al. (57) can be explicitly clarified as follows. Li et al. (57) employed C57BL/6 mice as the experimental model, exposing them to polystyrene microplastics (PS-MPs) via intraperitoneal injection. The study observed significant declines in skeletal muscle mass and grip strength, attributing these effects to dysregulation of the PI3K/Akt/mTOR signaling pathway, which is central to muscle protein synthesis and cell survival. This model directly demonstrates that microplastics interfere with intracellular signaling mechanisms to impair muscle function. While Amato-Lourenço et al. (56) primarily focused on human lung tissue and *in vitro* human bronchial epithelial cells (BEAS-2B), the study implicitly references prior research in rodent models (e.g., mice), where exposure to airborne microplastics induced alveolar inflammation and oxidative stress. These findings indirectly support the link between microplastic exposure and systemic effects on muscle health, as respiratory inflammation triggered by microplastics can propagate oxidative stress throughout the body. Together, these models—ranging from specific mouse strains to broader rodent studies—highlight how microplastics may compromise muscle function through both direct cellular interference and systemic inflammatory pathways. Thus, delving into the effects of microplastics on muscle functions and recovery processes is of great significance to evaluate the potential hazards of microplastics in the field of exercise physiology.

## 5 Microplastics and sports health risks

### 5.1 Cellular mechanisms of microplastic toxicity

Recently, the academic community has been increasingly focusing on the toxicological studies of microplastics, particularly on the potential effects these tiny particles may have on human health (Table 3). Research indicates that the core of the toxicity of microplastics lies in their destruction of cell architecture, specifically damage to key cellular components such as cell membranes, mitochondria, and endoplasmic reticulum (50). Such damage not only triggers cellular dysfunction, but can also trigger an inflammatory response, posing a potential threat to the organism health (58, 59). Moreover, microplastics possibly act as a carrier for other environmental pollutants, thus increasing the toxicity of these pollutants. For example, when microplastics are bound to harmful chemicals such as heavy metals and antibiotics, the biotoxic effects of these pollutants may be significantly strengthened, which further poses a higher risk to human health (60, 61). Some studies have focused on the toxic effects of microplastics on animal models, direct research on the effects of microplastics on human health is relatively insufficient, which highlights a significant gap in the assessment of microplastics for health risk. Thus, it is urgent to deepen toxicological studies to comprehensively and systematically assess the potential effects of microplastics on human health.

**Table 3:** Microplastics-induced sports health risks.

Category	Key points	Supporting evidence
Cellular mechanisms of microplastic toxicity	Destroys cell architecture (damage to cell membranes, mitochondria, endoplasmic reticulum). Triggers cellular dysfunction and inflammatory response. Acts as a carrier for other environmental pollutants (heavy metals, antibiotics), strengthening biotoxic effects.	(50, 58–61)
Long-term systemic health effects	Accumulates in the body and spreads to multiple organs via the blood circulatory system, causing systemic damage. Induces chronic inflammation, metabolic disorders, and long-term damage to immune system function. Changes microflora composition, increasing the risk of chronic diseases (cardiovascular diseases, diabetes).	(62–67).
Injury risks and epidemiological evidence	Compromises muscle function and respiratory efficiency, exacerbating injury susceptibility. Linked to reduced skeletal muscle mass in mice, increasing risk of muscle strains/tears during high-intensity activities. Endurance athletes exposed to airborne microplastics may experience reduced lung capacity, increasing fatigue-related injuries. Contact sport athletes face elevated microplastic exposure from turf infill materials, compounding injury risks (e.g., ACL injuries).	(44, 57, 68, 69)

### 5.2 Long-term systemic health effects

The potential health effects of long-term exposure to microplastics have not fully been understood, but scientific studies have provided evidence that long-term exposure to microplastics can cause various health problems (Table 3). Microplastics can not only accumulate in the body, but may even spread widely to multiple organs through the blood circulatory system, leading to systemic damage. Studies show that the long-term accumulation of microplastics may result in chronic inflammation, metabolic disorders, and long-term damage to the immune system function (62–65). Moreover, microplastics may also increase the risk of chronic diseases by changing the composition of the microflora and further affecting the metabolism and immune functions of the hosts. These diseases include, but are not limited to, cardiovascular diseases and diabetes (66, 67). For athletes and the general public, it is urgent to assess and monitor the long-term ingestion of microplastics for health risks. The aim is to protect public health by provide a basis for the development of scientific and effective prevention strategies and intervention plans. This problem is even more pressing for athletes who are continuously exposed to the environment. We expect to potential threats of microplastics to human health and promote the overall health and well-being of society by strengthening monitoring, delving into the potential impacts of microplastics, and taking targeted measures.

### 5.3 Injury risks and epidemiological evidence

As a core role in the field of sports health, epidemiological studies not only reveal the frequency and influencing factors of sports-related injuries but also need to integrate emerging environmental risks like microplastic exposure (Table 3). Research shows that athletes face specific injury risks not only from biomechanics but also from microplastic-induced physiological impairments. For example, microplastics absorbed via inhalation or diet may exacerbate injury susceptibility by compromising muscle function and respiratory efficiency. Studies have linked microplastic exposure to reduced skeletal muscle mass in C57BL/6 mice (57), which could directly increase the risk of muscle strains or tears during high-intensity activities. Additionally, endurance athletes exposed to airborne microplastics in synthetic turf environments may experience reduced lung capacity (44), limiting oxygen supply and increasing fatigue-related injuries.

Epidemiological evidence already highlights that contact sport athletes have higher awareness of anterior cruciate ligament (ACL) injuries (68), but emerging data suggest they may also face elevated microplastic exposure from turf infill materials. This dual risk—mechanical stress plus microplastic toxicity—warrants integrated analysis in injury surveillance. For instance, a 2024 study by Celik et al. noted insufficient participation in injury prevention training, which could be compounded if athletes remain unaware of microplastic-related risks. Future epidemiological studies should therefore prioritize mapping microplastic exposure gradients across sports (e.g., turf-based vs. natural surface disciplines) and correlating them with injury incidence, while developing preventive strategies that address both biomechanical and environmental stressors. This interdisciplinary approach will be critical for formulating comprehensive public health strategies in the era of pervasive microplastic pollution.

## 6 Summary and outlook

Microplastics in sports settings—primarily from synthetic turf infill (e.g., polystyrene particles), sportswear abrasion, and contaminated air/water—directly impact athlete physiological health and sports performance. Unlike traditional safety measures, controlling such microplastics is critical for protecting athletes. Research confirms their physiological harms: they reduce lung function (44), damage muscles by disrupting signaling pathways (57), impair endurance, prolong recovery, and increase injury risks. For example, polystyrene microplastics in artificial turf infill, a well-documented source (26), have these adverse effects.

To mitigate these impacts, targeted measures are needed. Policymakers should fund microplastic monitoring in training venues (14), promote eco-friendly alternatives like biodegradable turf infill, and restrict high-exposure activities such as training on synthetic turfs with high microplastic release rates during high-intensity sessions. For athletes, education on microplastic risks is key. When aware of links—such as how turf microplastics disrupt muscle signaling (57) or synthetic sportswear releases fibers during exercise (29)—they are 37% more likely to take protective actions (70). Coaches should teach athletes to identify sports-specific sources like inhaled turf particles in contact sports, microfibers from laundered synthetic fabrics, and ingestion via contaminated sports drinks (27), enabling them to choose low-pollution training sites or natural-fiber sportswear.

Current studies on microplastics and sports mainly focus on short-term effects in laboratories or animal models, with limited understanding of long-term impacts on athletes, who have high and frequent exposure. Thus, future research must focus on this group. Longitudinal epidemiological studies tracking athletes' microplastic exposure and health metrics (e.g., lung function, muscle recovery) are essential to clarify long-term risks and dose-response relationships.

Additionally, interdisciplinary research integrating sports science, environmental chemistry, and toxicology should explore how microplastic properties (size, composition) interact with exercise intensity to affect physiological functions, as well as their distribution and migration in sports environments. Based on these findings, develop microplastic-resistant sportswear and set exposure thresholds for sports facilities to protect athletes' physiological health and improve sports performance.
